# Engineering Work Function to Stabilize Metal Oxides in Reactive Hydrogen

**DOI:** 10.1021/acs.jpclett.4c03404

**Published:** 2025-03-03

**Authors:** Abdul Rehman, Robbert W. E. van de Kruijs, Wesley T. E. van den Beld, Jacobus M. Sturm, Marcelo Ackermann

**Affiliations:** Industrial Focus Group XUV Optics, MESA+ Institute for Nanotechnology, University of Twente, Drienerlolaan 5, 7522NB Enschede, The Netherlands

## Abstract

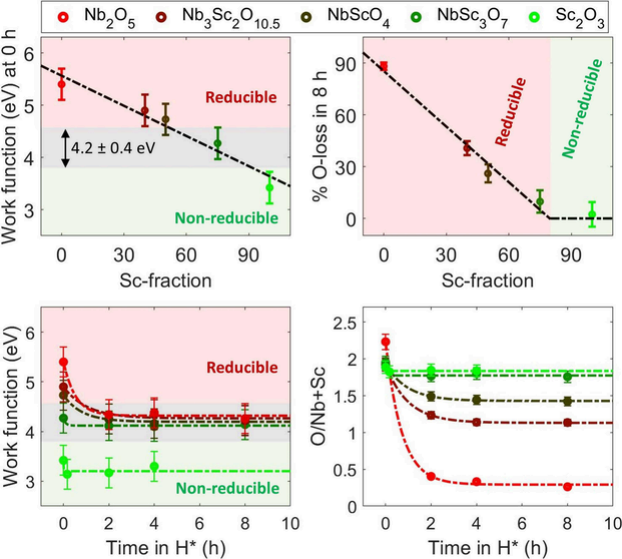

Hydrogen, crucial for the green energy transition, poses a challenge due to its tendency to degrade surrounding wall materials. To harness hydrogen’s potential, it is essential to identify the parameter(s) of materials that modulates hydrogen–material interaction. In a recent publication, we have shown that the reduction (denitridation) of transition metal (TM) nitrides in hydrogen radicals (H*) stops when their work function drops below a threshold limit. In this work, we tailor the work function of a complex TM oxide by tuning the relative contents of its constituent TM atoms. We show that increasing the fraction of a low-work function TM decreases the work function of the complex oxide, thereby decreasing its reducibility (deoxidation) in H*. This leads to the stabilization of the higher oxidation states of a high-work function TM, which otherwise would be readily reduced in H*. We propose that the work function serves as a tunable parameter, modulating the interaction of hydrogen with TM compounds.

Hydrogen plays a key role in a wide range of applications, from green energy solutions such as fusion,^1^ energy storage,^2,3^ and transport^4^ to advanced semiconductor fabrication, where it serves as an etchant,^5,6^ and a reducing agent.^7,8^ Nevertheless, the tendency of hydrogen to react with and diffuse into the surrounding wall materials poses a high operational risk, e.g., embrittlement, blistering, interface defects, and chemical erosion.^9−14^ Hence, to fully realize hydrogen’s potential, it is essential to develop novel coatings that are stable in reactive hydrogen environments and can protect hydrogen-sensitive system components. The development of such coatings necessitates a strategic approach to effectively modulate the interaction between materials and hydrogen.

In recent publications,^15,16^ we demonstrate that the chemical stability of transition metal nitrides (TMNs) in high-temperature hydrogen radical (H*) environments depends on their work function. When the work function of a TMN system drops below a threshold value (ϕ_TH_ = 4.3 ± 0.4 eV, in H* at increased temperatures), its reduction (denitridation) effectively stops, even though further reduction remains thermodynamically favorable, i.e., a negative change in the Gibbs free energy (Δ*G*) for the reduction reaction: TMN_*y*_ + *x*H → TMN_*y*–1_ + NH_*x*_. We explain this by the preferential binding of H* to transition metal (TM) atoms,^17,18^ which impedes the formation of volatile NH_*x*_ species.

In this work, we demonstrate that the work function serves as a tunable parameter, enabling control over the reduction (deoxidation) of (complex) TM oxides in H*. By strategically alloying a high-work function oxide with a lower-work function oxide, we show that the work function of the resulting complex oxide can be modulated by tuning the relative fraction of its constituent TM atoms. This approach aligns with the literature reported for binary metal alloy systems.^19−21^ The shift in the work function correlates directly with the reduction of the complex oxide in H*, with a lower work function leading to a lower reduction. Furthermore, we show that the higher oxidation states of a high-work function TM in the complex oxide are stable in H*, which otherwise would be reduced readily.

For our study, we selected Nb_2_O_5_, Sc_2_O_3_, and their complex oxides (NbSc_*y*_O_*x*_). Nb_2_O_5_ and Sc_2_O_3_ present extreme cases in our study. Nb_2_O_5_ has a high work function of ≈5.2 eV,^22^ which, according to our work function model,^15^ is expected to undergo significant reduction during H* exposure. In contrast, Sc_2_O_3_ due to its lower work function (≈3.5 eV^23^) is expected to be nonreducible in H*.

Given that the formation of NbSc_*y*_O_*x*_ (solid solution) is energetically feasible,^24,25^ we can modulate its local work function by altering the relative proportions of Nb and Sc atoms. For this study, we specifically chose NbSc_*y*_O_*x*_ compositions with approximately 40%, 50%, and 75% Sc atoms relative to Nb atoms, aiming to vary the work function of NbSc_*y*_O_*x*_ around ϕ_TH_.^15^ As a starting point, we estimated the work function of NbSc_*y*_O_*x*_ using a compositional weighted average of the work functions of its constituent TM oxides.^21^ We recognize that this estimation does not take into account critical factors such as surface termination, orientation, and unique heterostructuring in complex oxides, all of which influence the actual work function.^26^ Nevertheless, this estimation provides a baseline for approximating the trend in the work function of a complex oxide as the relative proportion of its constituent TM atoms is varied.

We exposed 5 ± 0.5 nm thin films of the selected materials to H* at 550 °C for a total duration of 8 h. These H*-exposure conditions are relevant for the development of hydrogen-protective coatings for EUV scanners and fusion reactors.^13,27−29^ The angle-resolved X-ray photoelectron spectroscopy (AR-XPS) measurements are performed on the samples after H* exposure for 2, 4, and 8 h. Since the Sc_2_O_3_ sample is effectively nonreducible, it is exposed to H* for only 4 h, with AR-XPS measurements performed after 10 min, 2 h, and 4 h. To saturate thermally induced processes prior to high-temperature H* exposures, the samples are annealed in a vacuum for 2 h at 550 °C. These samples, termed pre-exposed (0 h) in the text and figures, are used as a reference to assess the reducibility of the oxides. The samples exposed to H* are denoted by their total H*-exposure times: exposed to H* for 10 min, 2 h, 4 h, and 8 h. The stoichiometry/spectra of the samples mentioned in the text and figures correspond to the XPS measurements taken at a takeoff angle (Θ) of 34.25°. Note that work function (surface average) measurements are also performed via XPS in normal lens mode.^15^

In the subsequent paragraphs, we first discuss that as the Sc fraction in NbSc_*y*_O_*x*_ increases, both its work function and reduction decrease. Next, we discuss that this decreased reduction leads to the stabilization of higher oxidation states of Nb, where these oxidation states in Nb_2_O_5_ are readily reduced.

In line with our model,^15^ the Nb_2_O_5_ sample with a high work function undergoes strong reduction upon H* exposure (Figure 1). The strong reduction of the sample is evident from the pronounced decrease in the O/Nb ratio (Figure 1b,d). Inversely, the Sc_2_O_3_ sample is effectively nonreducible due to its low work function. A slight decrease in the O/Sc ratio upon exposure to H* for 10 min is attributed to the etching of the surface adventitious carbon layer present on the pre-exposed sample, which contains O atoms (Figure 1 and Figure S13). Note that the O/Sc ratio in the Sc_2_O_3_ sample is approximately 1.9 (Figure 1d). The high O fraction in the sample is attributed to the formation of ScOOH (Figure 3).^30−32^

**Figure 1:**
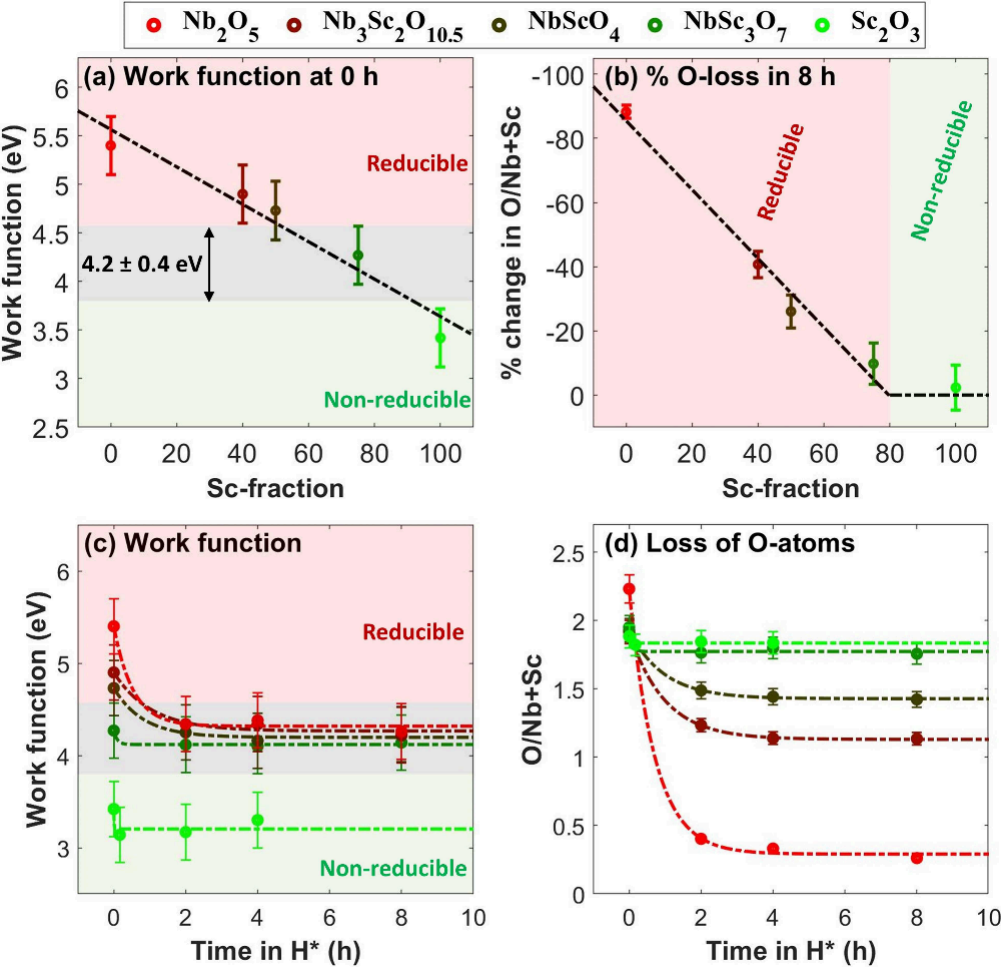
Measured work functions, % O loss, and O/Nb+Sc ratios in the Nb_2_O_5_, Nb_3_Sc_2_O_10.5_, NbScO_4_, NbSc_3_O_7_, and Sc_2_O_3_ samples. (a) The work function of the pre-exposed (0 h) samples decreases almost linearly with an increase in the Sc fraction. (b) This decrease in the work function leads to a decrease in the O loss in the samples upon H* exposure, calculated as the percent change in the O/Nb+Sc ratio. . (c and d) As O atoms are removed from the samples during H* exposure, their work function progressively decreases, until reaching a stable value of 4.2 ± 0.4 eV. At this threshold (ϕ_TH_), the reduction reaction effectively stops.

The work function of the NbSc_*y*_O_*x*_ samples exhibits a clear dependence on the Sc fraction. Notably, the work function of the pre-exposed (0 h) NbSc_*y*_O_*x*_ samples decreases almost linearly with an increase in Sc fraction, consistent with our estimation (Figure 1a). Furthermore, the offset between the measured and linearly approximated work functions is a couple tenths of an eV. This agreement may be attributed to the amorphous/nanocrystalline morphology of the samples (Figures S1 and S3), which could minimize the impact of unique surface terminations, orientations, and heterostructuring that might be present in the complex oxide phase.

The decrease in the work function with an increase in Sc fraction is correlated with a smaller decrease in the O/Nb+Sc ratios upon H* exposure (Figure 1a,b). Specifically, the change in both the work function and the O/Nb+Sc ratio upon H* exposure is smaller for the samples with a higher Sc fraction (Figure 1c,d). This indicates that the extent of oxide reduction in H* is directly related to its work function, i.e., decreasing with a decreasing work function.

Notably, the reduction reaction of all of the samples effectively stops as their work function decreases to 4.2 ± 0.4 eV (Figure 1c,d), aligning with our model.^15^ Since a sufficient number of O atoms remain at the surface level following the last H* exposure (Figures S4, S5b–S7b, and S8), the reduction reaction is not limited by the diffusion of subsurface O atoms to the surface.^15^ Therefore, based on the modeling by Van de Walle et al.,^17,18^ we propose that when the work function of an oxide system is higher than 4.2 ± 0.4 eV, H* adsorption on O atoms is favorable, enabling the formation of volatile OH_*x*_ species.^15^ However, as electronegative atoms (in this case, O atoms) are removed^33^ or electropositive atoms (in this case, Sc atoms) are incorporated, the work function of the oxide system decreases, eventually reaching the 4.2 ± 0.4 eV threshold limit (Figure 1b). At this point, H* preferentially binds to TM atoms instead of O atoms, making the formation of OH_*x*_ unfavorable.

Due to the work function threshold limit, the pre-exposed samples with a lower work function exhibit a higher O/Nb+Sc ratio following the last H* exposure (Figure 1d), suggesting that higher oxidation states of Nb and Sc atoms are stabilized with an increase in Sc fraction. We discuss the Nb 3d and Sc 2p XPS spectra of the samples in the subsequent paragraphs, which provide insights into the oxidation states of Nb and Sc atoms in the NbSc_*y*_O_*x*_ samples.

In all pre-exposed samples, Nb atoms are in the +5 oxidation state, except in Nb_2_O_5_, where a minor fraction of Nb atoms exhibit a +4 – δ oxidation state (Figure 2a). After exposure to H* for 8 h, the oxidation states of Nb atoms depend on the Sc fraction (Figure 2b). The Nb atoms in the samples with a higher Sc fraction exhibit a higher fraction of higher oxidation states after exposure to H* for 8 h. For instance, in the Nb_2_O_5_ sample, ≈28% of Nb atoms are in a +2 – δ oxidation state(s), with the remainder being metallic (Nb°). In comparison, the samples with approximately 40%, 50%, and 75% Sc show approximately 10%, 17%, and 51% Nb atoms, respectively, in a +5 oxidation state. The rest of the Nb atoms in these samples are distributed among +2 ± δ oxidation states (Figure 2b). The presence of multiple oxidation states of Nb atoms in the H*-exposed samples is attributed to the understoichiometric O/Nb ratio, due to which O atoms are distributed among Nb atoms in such a way that minimizes the formation energy. Multiple oxidation states lead to a non-uniform distribution of the local work function.^33^ According to our model,^15^ this variation in the local work function influences the reduction process, resulting in Nb atoms in higher oxidation states undergoing preferential reduction over those in lower oxidation states.

**Figure 2:**
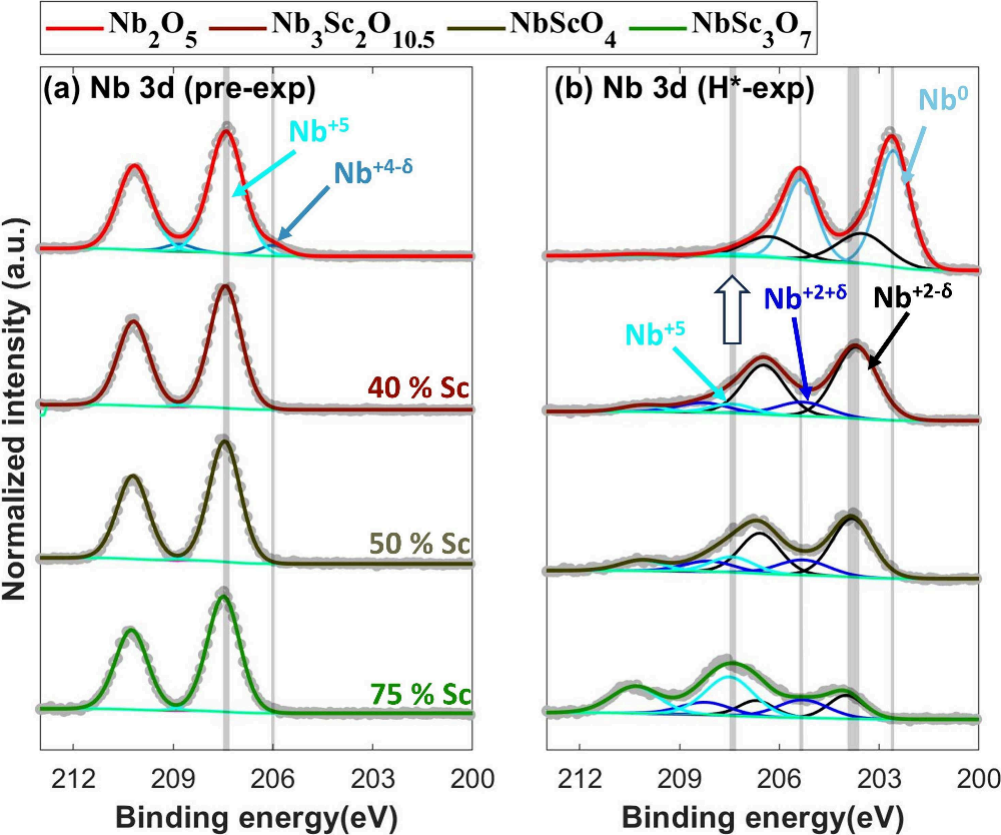
Nb 3d XPS spectra of (a) the pre-exposed and (b) the 8 h H*-exposed Nb_2_O_5_, Nb_3_Sc_2_O_10.5_, NbScO_4_, and NbSc_3_O_7_ samples. In the pre-exposed samples, Nb atoms are predominantly in a +5 oxidation state. The oxidation states of Nb atoms in the post-H*-exposure samples depend on the Sc fraction in NbSc_*y*_O_*x*_. A higher Sc fraction results in a greater proportion of Nb atoms remaining in higher oxidation states after H* exposure. This suggests that the samples predominantly contain NbSc_*y*_O_*x*_, rather than distinct NbO_*x*_ and ScO_*x*_ phases.

The stabilization of higher oxidation states of Nb atoms with an increase in the Sc atom fraction in NbSc_*y*_O_*x*_ suggests that the incorporation of Sc atoms (low work function) can effectively decrease the reducibility of Nb_2_O_5_ (higher work function) in H*. This is analogous to the decreasing reducibility of Nb_2_O_5_ as O atoms are removed^33^ (Figure 1c,d).

Unlike the Nb atoms, no change in the oxidation state of the Sc atoms is observed upon H* exposure. In both the pre- and post-H*-exposure samples, the Sc atoms are in a +3 oxidation state (Figure 3). According to the literature, Nb has various oxidation states that are stable in bulk form,^34−36^ while Sc is predominantly found in a +3 oxidation state.^30−32^ Since the O/Sc ratio exceeds 1.5 in all samples and Sc has a higher oxidation potential than Nb, Sc atoms retain their +3 oxidation state upon H* exposure. Furthermore, variation in the coordination of O atoms with Nb and Sc atoms within the complex oxide phase may lead to differences in the electronic environment across the unit cell. This could also result in O atoms more closely coordinated with Nb atoms being preferentially removed during H* exposure.

**Figure 3:**
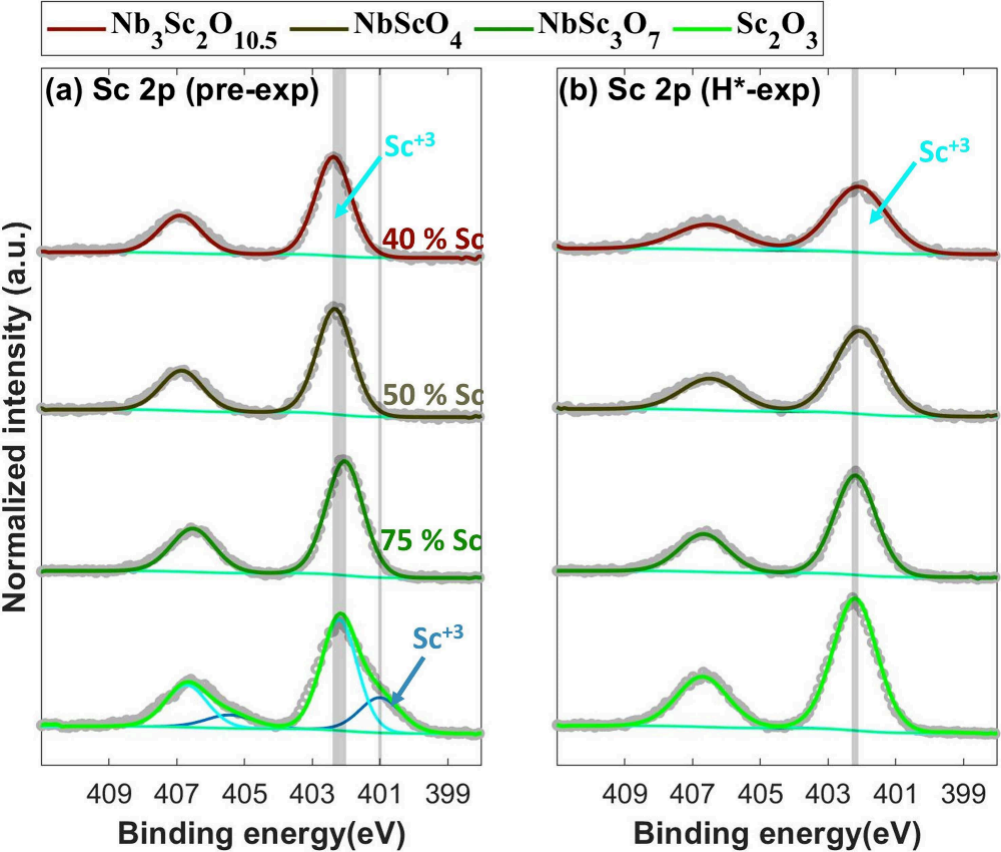
Sc 2p XPS spectra of the (a) pre-exposed and (b) 8 h H*-exposed Nb_3_Sc_2_O_10.5_, NbScO_4_, and NbSc_3_O_7_ samples, along with 4 h H*-exposed Sc_2_O_3_ samples. In all of the samples, Sc atoms are in a +3 oxidation state, with the Sc_2_O_3_ doublet appearing at a lower binding energy and the ScOOH doublet at a higher binding energy. For the samples where O loss is substantial (Nb_3_Sc_2_O_10.5_ and NbScO_4_), the Sc 2p spectra show significant changes upon H* exposure. No significant change in the Sc 2p spectra of the NbSc_3_O_7_ sample is observed. The changes in the Sc 2p spectra of the Sc_2_O_3_ sample are attributed to hydrogenation upon H* exposure.

While the oxidation state of Sc atoms remains unchanged, in the samples where substantial O loss occurred during H* exposure, changes in the Sc 2p spectra are observed. For instance, in the samples with approximately 40% and 50% Sc, the Sc^3+^ doublet shifted by ≈0.3 eV toward a lower binding energy upon exposure to H* for 8 h. Furthermore, the full width at half-maximum (FWHW) of the fitted doublet is increased by ≈57% and ≈37%, respectively, compared to the pre-exposed sample (Figure 3). This indicates an O loss during H* exposure. In contrast, in the sample with 75% Sc, where the O/Nb+Sc ratio did not decrease significantly, no major change in the Sc 2p spectra is noted. The change in the Sc 2p spectra of the Sc_2_O_3_ sample after H* exposure is due to the hydrogenation of the sample (Figure 3).^30^ Overall, the evolution of the Sc 2p XPS spectra with the Nb fraction suggests that the incorporation of Nb atoms into Sc_2_O_3_ influences its interaction with H*.

The stabilization of Nb^5+^ (Figure 2) with an increase in the Sc fraction suggests that the samples predominantly contain a NbSc_*y*_O_*x*_ solid solution. This is because the reducibility of a TM compound is governed by its local work function.^15^ If the samples were primarily composed of separate NbO_*x*_ and ScO_*x*_ phases, the local work function of the NbO_*x*_ phase would remain unchanged regardless of the Sc fraction in the sample. Consequently, the reducibility of Nb would also be unaffected by the Sc fraction. Further evidence supporting this conclusion comes from the low-kinetic energy (LKE) XPS spectra (Figure S14), which show no surface phases with significantly different work functions, as would be expected if NbO_*x*_ and ScO_*x*_ phases were present. Similarly, kelvin probe atomic force microscopy (KPAFM) (Figure S3) detects no significant variation in the work function across the surface with a spatial resolution of approximately 100 nm. Furthermore, transmission electron microscopy energy-dispersive X-ray spectroscopy (TEM-EDS) analysis confirms the homogeneous distribution of TM and O atoms across the sample’s depth (Figure S2).

Our results demonstrate that alloying NbO_*x*_ and ScO_*x*_ predominantly results in the formation of NbSc_*y*_O_*x*_ (complex oxides), with the reduction of NbSc_*y*_O_*x*_ in H* being dependent on the Nb/Sc ratio. As the Sc fraction increases, the work function of NbSc_*y*_O_*x*_ decreases. This decrease in the work function leads to a decrease in the reduction of NbSc_*y*_O_*x*_, thereby stabilizing the higher oxidation states of Nb atoms. Additionally, this study shows that the reduction reaction on all of the studied oxide systems stops when their work function reaches a 4.2 ± 0.4 eV threshold, consistent with our recent publication.^15^

Based on our findings, we propose that the work function of a TM compound serves as a predictive tunable parameter, governing its chemical stability in reactive hydrogen environments. Decreasing the work function effectively decreases the TM compound’s reducibility. Furthermore, our work demonstrates that by modulating the work function specific oxidation states of a TM can be stabilized in a hydrogen environment. These insights provide a valuable framework for designing TM compound hydrogen-protective coatings by strategically engineering their work function.

## Methodology

Thin films of Nb_2_O_5_, Nb_3_Sc_2_O_10.5_, NbScO_4_, NbSc_3_O_7_, and Sc_2_O_3_ are deposited onto Si(100) substrates through reactive DC magnetron co-sputtering using Nb and Sc targets. The deposition chamber maintains a base pressure in the low 10^–8^ mbar range. Ar (99.999%) and O_2_ (99.999%) with flow rates of 20 sccm are used as sputtering gases, providing a working pressure of 1 × 10^–3^ mbar. The deposition rates of Nb_2_O_5_ and Sc_2_O_3_ were calibrated as a function of the magnetron currents. Approximately 20 nm thick films were deposited for calibrations. Film thicknesses are measured using X-ray reflectivity (XRR), performed using a Malvern Panalytical Empyrean laboratory diffractometer, which employs a monochromatic Cu Kα1 radiation source. The magnetron currents are then adjusted to achieve approximately 40%, 50%, and 75% Sc atoms relative to Nb atoms in the final samples.

The 5 ± 0.5 nm thin films are deposited, where the thickness is controlled by the deposition time (Table S1). The thickness of the sample is chosen to ensure that the entire depth of the samples is probed by angle-resolved X-ray photoelectron spectroscopy (AR-XPS). XPS measurements are performed using a Thermo-Fisher theta probe angle-resolved X-ray photoelectron spectrometer, which utilizes a monochromatic Al Kα radiation source. During AR-XPS measurements, spectra are collected at takeoff angles (Θ, from the sample’s surface normal) ranging from 26.75° to 71.75°, providing probing depths ranging from ≈5 to ≈1.5 nm, respectively, with a spot size of 400 μm × 400 μm. Note that for quantification, we calibrated AR-XPS signals based on the transmission function measured for the normal (not angle-resolved) lens mode. Furthermore, the stoichiometry of the samples mentioned in the text and figures is measured at Θ = 34.25°, with an uncertainty of ±10%.

**Figure 4:**
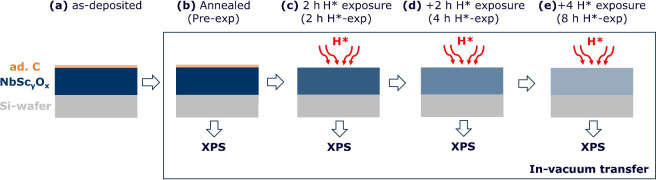
Schematic of the methodology. (a) NbSc_*y*_O_*x*_ samples are deposited via reactive DC magnetron co-sputtering. A thin layer of adventitious carbon (ad. C) formed on the samples’ surfaces during ambient storage. (b) The samples are first annealed at 550 °C for 2 h. (c–e) The samples are then exposed to H* for a total of 8 h at 550 °C, where XPS measurements are performed on the samples after (c) 2, (d) 4, and (e) 8 h. The samples are transferred in vacuum (lower range of 10^–9^ mbar) between the processing (annealing/H* exposure) and XPS chambers.

After deposition, samples are stored under ambient conditions for approximately one week, during which they accumulate adventitious carbon (ad. C). The presence of O atoms in the ad. C layer slightly affects (increases) the calculated O/Nb+Sc ratio in the pre-exposed samples (Figure 4a). The samples stored under ambient conditions are then vacuum annealed (Figure 4b) in the processing chamber at 550 °C for 2 h to saturate thermally induced processes before being exposed to H* in the same chamber. The base pressure of the processing chamber is in the 10^–8^ mbar range, while during annealing, the maximum pressure of the chamber is in the 10^–7^ mbar range. The temperature of the sample is measured using an N-type thermocouple, which is clamped on the sample surface. After annealing, the samples are cooled to approximately 100 °C and then transferred to the XPS chamber through a vacuum with a pressure in the 10^–9^ mbar range. The XPS measurements on the annealed samples are denoted as pre-exposed (pre-exp) in the text and figures. The measurements on the pre-exposed samples are used as the reference for assessing the reduction of the samples upon H* exposure.

The pre-exposed samples are then transferred through a vacuum back to the processing chamber for H* exposure. H* atoms in the chamber are generated through thermally cracking H_2_ via a W filament heated to ≈2000 °C. For H* exposure, the working pressure of the chamber is set to 0.02 mbar and the samples are placed ≈0.05 m from the W filament. The H* flux corresponding to these settings is calculated to be 10^21±1^ H* m^–2^ s^–1^.^16^ During H* exposure, the sample temperature is maintained at 550 °C. These H*-exposure conditions are relevant to fusion reactors and EUV scanners.^13,27−29^

Except for the Sc_2_O_3_ sample, the samples are exposed to H* for a total of 8 h, with XPS measurements taken after 2 h (Figure 4c), 4 h (Figure 4d), and 8 h (Figure 4e). The Sc_2_O_3_ sample is exposed to H* for a total of 4 h, with XPS measurements taken after 10 min, 2 h, and 4 h. The XPS measurements on the H*-exposed samples are labeled according to the total H*-exposure time, i.e., 10 min H*-exp, 2 h H*-exp, 4 h H*-exp, and 8 h H*-exp. Note that before each XPS measurement, the samples are cooled to 100 °C in the processing chamber and then transferred in vacuum to the XPS chamber.

The reducibility of the samples is evaluated based on the decrease in the ratio of the atom % of O to the atom % of Nb+Sc (O/Nb+Sc) upon H* exposure. The O, Nb, and Sc fractions in the samples are measured by effectively integrating the intensities (after background subtraction, calculated via the Shirley method) of their respective XPS spectra (O 1s, Nb 3d, and Sc 2p, respectively). These intensities are then scaled according to their respective Scofield sensitivity factors.^37^ The decrease in the O/Nb+Sc ratios as a function of H*-exposure time, along with the changes in the Nb 3d and Sc 2p XPS spectra taken at Θ = 34.25°, is discussed in the text. Note that Nb 3d and Sc 2p XPS spectra presented in the main text figures are fitted with Voigt profile doublets, following Shirley background subtraction (the fitting method is detailed in the Supporting Information). Furthermore, for better visualization, Nb 3d and Sc 2p XPS spectra are normalized to the maximum intensity of Nb 3d and Sc 2p spectra of the pre-exposed samples, respectively.

Based on AR-XPS measurements, the chemical composition across the depth of the samples is found to be homogeneous. Variation in the O/Nb+Sc and Sc/Nb ratios as a function of Θ for each AR-XPS measurement is less than 10% (Figures S4–S8). Therefore, we discuss only the XPS spectra taken at Θ = 34.25° in the main text. The homogeneity of the pre-exposed NbScO_4_ sample across the depth is further confirmed by cross-sectional transmission electron microscopy (TEM) with energy-dispersive X-ray spectroscopy (EDS) (Figure S2). The Supporting Information further contains as-collected Nb 3d, Sc 2p, O 1s, and Si 2p XPS spectra as a function of Θ for each AR-XPS measurement (Figures S9–S13).

The work function of the samples is measured via XPS.^15^ To do this, we collect both the low-kinetic energy (LKE) and valence band (VB) spectra at a negative bias of 16.4 V. This bias accelerates low-kinetic energy (secondary) electrons from the sample toward the analyzer and separates these electrons from those scattering off the analyzer’s wall. To assess whether the samples accumulate a net charge during measurements, particularly given the high dielectric constants of oxides, we also collect VB spectra of the samples without bias. The offset in the binding energies of the VB spectra collected with and without bias is almost equal to the applied bias. This suggests that our samples exhibit sufficient conductivity (Figures S14 and S15). Furthermore, LKE spectra exhibit a single secondary electron cutoff, suggesting that variation in the work function across the surface is insignificant (Figure S14).^38^ Note that, in our setup, the electron analyzer and the sample’s normal are not aligned, which introduces a systematic offset of −1 ± 0.2 eV in the measured work function.^15^ The work function values reported in the text and figures have already been adjusted to account for this offset. Furthermore, the uncertainty in measuring the secondary electron cutoff is ±0.1 eV, resulting in an uncertainty of ±0.2 eV in the difference between the measured work function values.

To validate the work function measurements, we also measure the work function of the pre-exposed Nb_2_O_5_ and NbScO_4_ samples using KPAFM. These work function measurements are in close agreement with those obtained by using XPS (Figure S16). Furthermore, KPAFM measurements also suggest that the work function variation across the samples’ surfaces is insignificant, consistent with the LKE XPS spectra.

## Supporting Information Available

XRD of the pre-exposed samples, cross-sectional TEM images of the pre-exposed NbScO_4_ sample, KPAFM measurements of the pre-exposed Nb_2_O_5_ and NbScO_4_ samples, calculated Sc/Nb and O/Nb+Sc ratios as a function of Θ, fitting method for the Nb 3d and Sc 2p XPS spectra, comparison of the Nb 3d, Sc 2p, O 1s, and Si 2p XPS spectra as a function of Θ, and LKE and VB spectra of the samples (PDF)
