# Supplementary material for: Engineering Work Function to Stabilize Metal Oxides in Reactive Hydrogen
Source: J Phys Chem Lett. 2025 Mar 3;16(10):2592–8. doi: 10.1021/acs.jpclett.4c03404 (PMC11912526; doi:10.1021/acs.jpclett.4c03404)
Supplement: Supplementary file 1 — jz4c03404_si_001.pdf [file jz4c03404_si_001.pdf]

# Supporting Information
# Engineering Work Function to Stabilize Metal Oxides in Reactive Hydrogen

Abdul Rehman[†*], Robbert W.E. van de Kruijs[†], Wesley T.E. van den Beld[†], Jacobus M. Sturm[†], and Marcelo Ackermann[†]

[†]*Industrial Focus Group XUV Optics, MESA+ Institute for Nanotechnology, University of Twente, Drienerlolaan 5, 7522NB Enschede, the Netherlands*

* E-mail: a.rehman@utwente.nl

# Content

| | |
|---|---|
| X-ray Diffraction | S3 |
| Cross-Sectional Transmission Electron Microscopy | S3 |
| Contact Potential Difference | S4 |
| Nb/Sc and O/Nb+Sc ratios as the Function of $\Theta$ | S4 |
| Nb 3d and Sc 2p XPS spectra fitting | S7 |
| Comparison of the X-ray Photoelectron Spectroscopy spectra as a function of $\Theta$ | S9 |
| Low Kinetic Energy and Valance Band XPS Spectra | S12 |
| Work Function measurements via KPAFM | S13 |
| Deposition rates of the samples | S14 |
| Bibliography | S15 |

# X-ray Diffraction

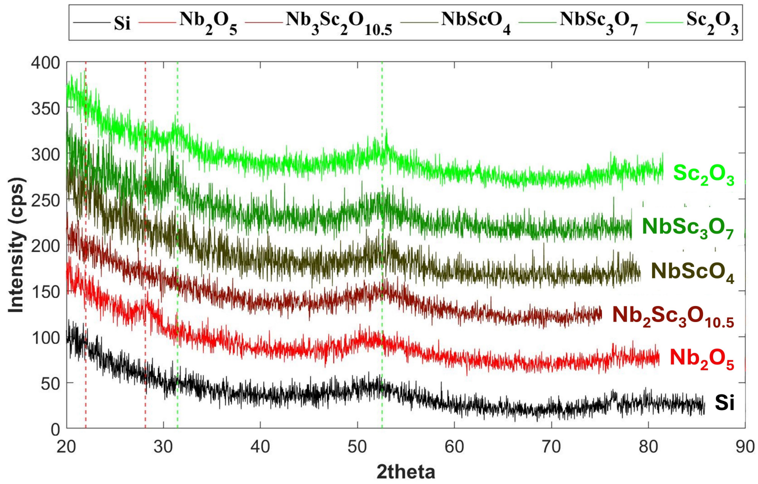


**Figure S1.** Grazing incidence X-ray diffraction (GIXRD) of the pre-exposed samples (omega fixed at 0.8°). The $Nb_2O_5$, $Sc_2O_3$, and $NbSc_3O_7$ samples each showed only a single peak, making definitive identification of their crystal phases uncertain. Overall, the GIXRD results suggest that the samples have nanocrystalline/amorphous morphology.

# Cross-Sectional Transmission Electron Microscopy

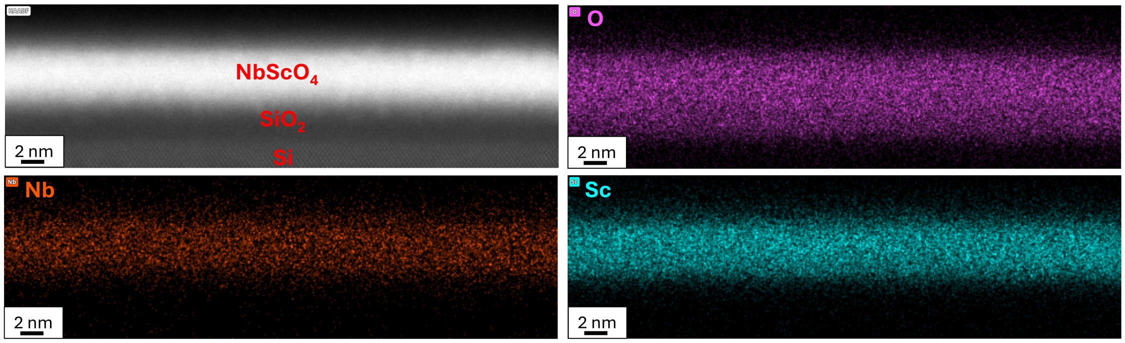


**Figure S2.** Cross-sectional Transmission Electron Microscopy (TEM) and Energy Dispersive X-ray Spectroscopy (EDS) of the pre-exposed $NbScO_4$ sample. The sample appears amorphous, consistent with GIXRD (Figure S1). O-, Nb-, and Sc-atoms are homogeneously distributed across the sample's depth, in line with the AR-XPS measurement (Figure S6). The homogeneous distribution of O-, Nb-, and Sc-atoms suggests the absence of separate $NbO_x$ and $ScO_x$ phases across the sample's depth.

# Contact Potential Difference

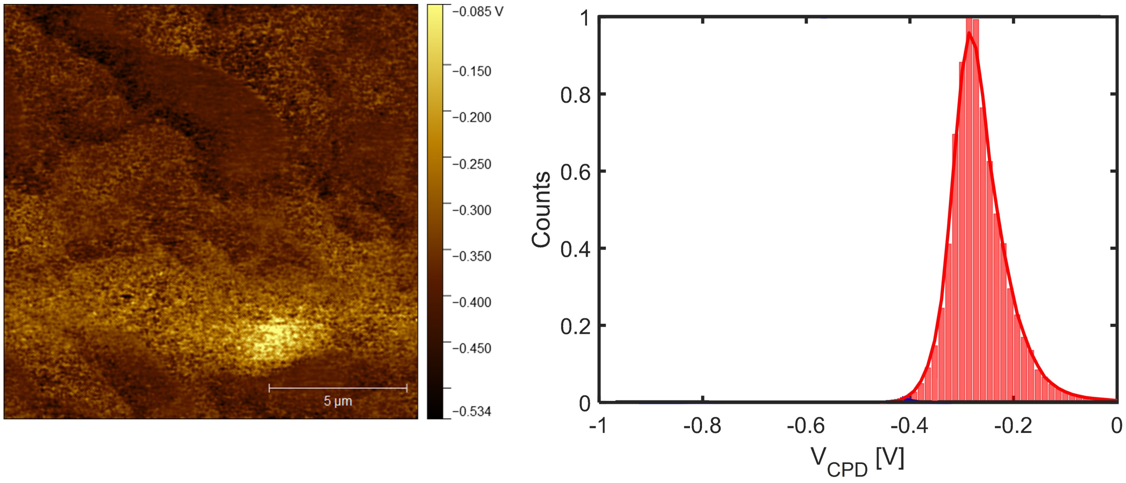


**Figure S3.** Contact potential difference ($V_{CPD}$) across the surface of the pre-exposed $NbScO_4$ sample measured via Kelvin Probe Atomic Force Microscope (KPAFM). The variation in the $V_{CPD}$ is insignificant, suggesting the absence of separate $NbO_x$ and $ScO_x$ phases across the samples surface, consistent with the Low Kinetic Energy (LKE) XPS spectra of the sample (Figure S14e).

# Nb/Sc and O/Nb+Sc ratios as the Function of Θ

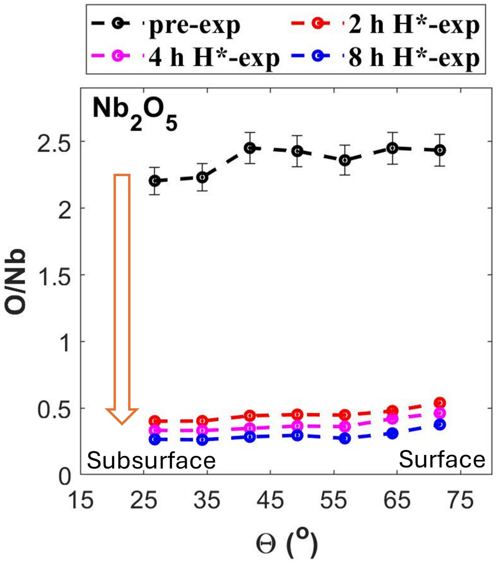


**Figure S4.** O/Nb ratio in the $Nb_2O_5$ sample measured as a function of Θ. The ratio decreases uniformly with Θ upon H*-exposure.

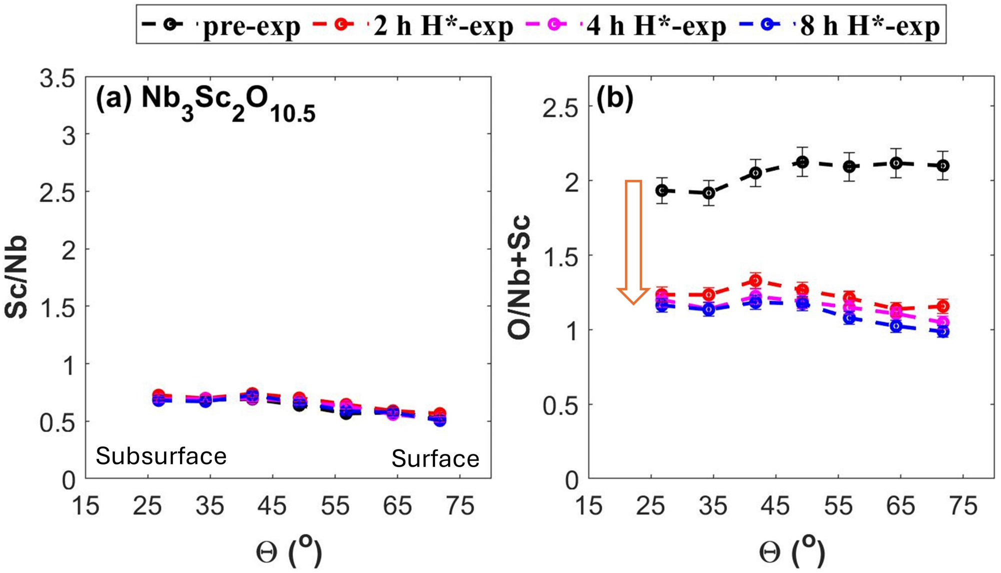


**Figure S5.** (a) Sc/Nb and (b) O/Nb+Sc ratios in the $Nb_3Sc_2O_{10.5}$ sample measured as a function of Θ. The distribution of Nb- and Sc-atoms remains unchanged upon H*-exposure. The decrease in the O/Nb+Sc ratio upon H*-exposure is less than that observed in the $Nb_2O_5$ sample (Figure S4).

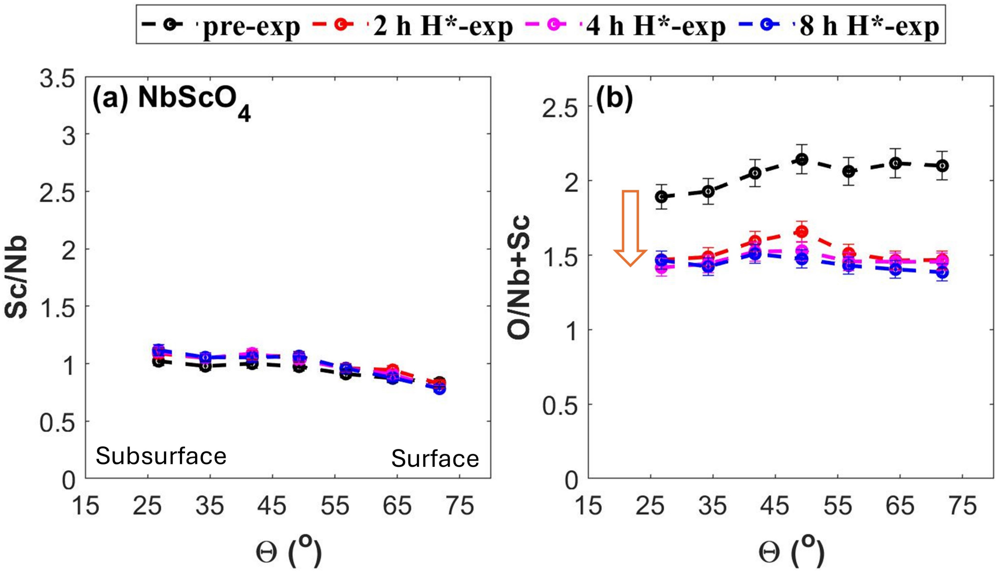


**Figure S6.** (a) Sc/Nb and (b) O/Nb+Sc ratios in the $NbScO_4$ sample measured as a function of Θ. There is no significant change in the distribution of Nb- and Sc-atoms upon H*-exposure. Furthermore, the oxide reduction is less pronounced than in the $Nb_3Sc_2O_{10.5}$ and $Nb_2O_5$ samples (Figure S4 and S5).

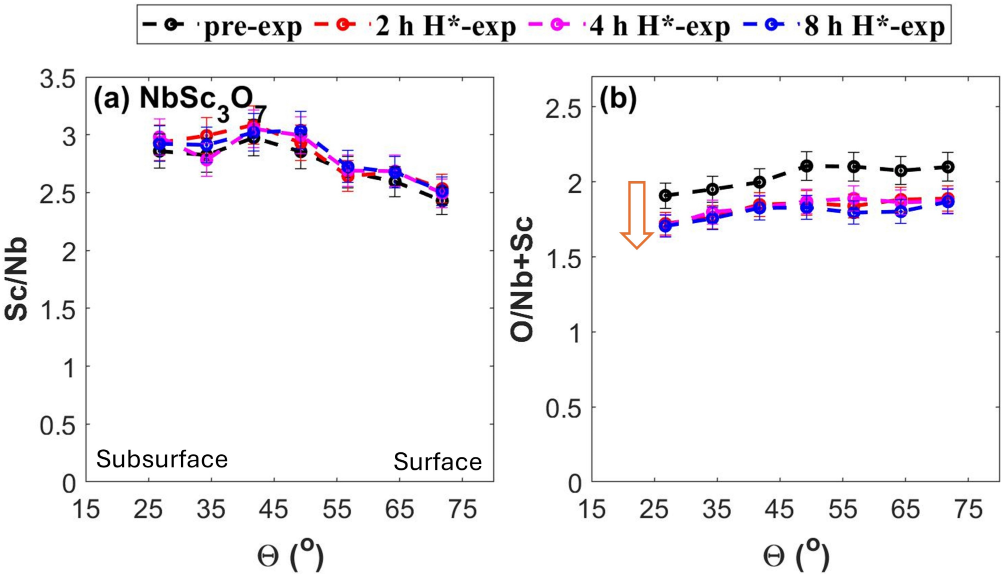


**Figure S7.** (a) Sc/Nb and (b) O/Nb+Sc ratios in the $NbSc_3O_7$ sample measured as a function of Θ. The Sc-fraction at the surface level is slightly lower than in the subsurface region. The reduction of the sample upon H*-exposure is minimal due to its low work function.

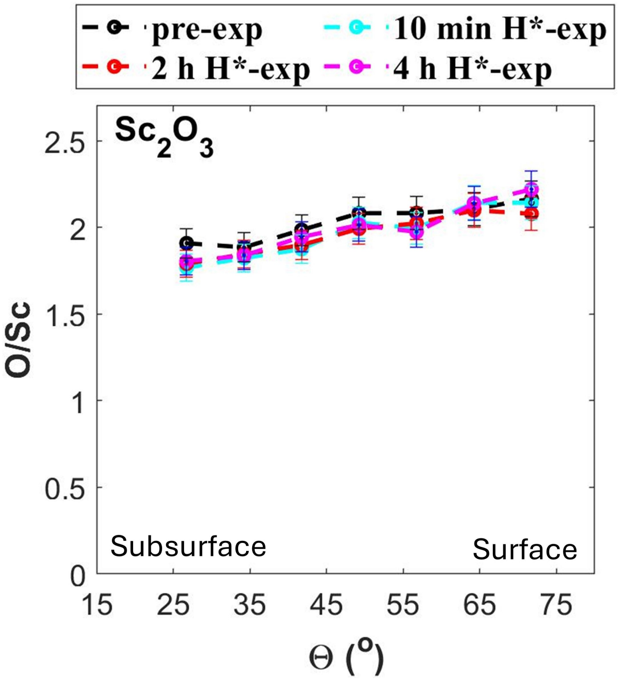


**Figure S8.** O/Sc ratio in the $Sc_2O_3$ sample measured as a function of Θ. The O/Sc ratio remains unchanged upon H*-exposure, indicating that the sample is effectively non-reducible. The higher O/Sc ratio at the surface level is attributed to the formation of ScOOH.

# Nb 3d and Sc 2p XPS spectra fitting

In this section, we outline the methodology used for deconvoluting the Nb 3d and Sc 2p XPS spectra.

For all XPS spectra fitting, we applied Shirley background subtraction. The spectra were then fitted using Voigt profile doublets with Gaussian-Lorentzian mixing % fixed at 30. The following paragraphs detail the constraints; binding energy ($E_B$), full width at half maximum (FWHM), and height ratio and separation between components of the doublets used in the fitting process.

## Nb 3d XPS spectra fitting

For the Nb 3d spectra, the height and FWHM ratio between doublet components (Nb $3d_{3/2}$/Nb $3d_{5/2}$) were fixed at 0.689 and 1, respectively, with a $E_B$ separation of 2.8 $\pm$ 0.2 eV.

All the pre-exposed samples, except for the $Nb_2O_5$, were fitted with single doublet, with Nb $3d_{5/2}$ at 207.4 $\pm$ 0.1 eV $E_B$ and FWHM of 1.2 $\pm$ 0.1 eV. This doublet corresponds to Nb-atoms in +5 oxidation state.[1]

In the pre-exposed $Nb_2O_5$ sample, an additional doublet was fitted with Nb $3d_{5/2}$ at 206 eV $E_B$ and FWHM of 1 eV. This second doublet represents Nb-atoms in a $+4-\delta$ oxidation state.[1–3]

Nb 3d spectrum of the 8 h H*-exposed $Nb_2O_5$ sample was fitted with two doublets. The $E_B$ and FWHM were left as free parameters. Nb $3d_{5/2}$ components of the doublets are fitted at 202.6 eV and 203.5 eV $E_B$, with FWHM of 1.2 eV and 1.9 eV, respectively. These doublets are assigned to Nb-atoms in 0 and $+2-\delta$ oxidation states, respectively.[1–3]

The Nb 3d spectra of the sample containing Sc-fraction were fitted with three doublets, corresponding to Nb-atoms in +5, $+2+\delta$, and $+2-\delta$ oxidation states. The $E_B$ of the Nb $3d_{5/2}$ components of the +5 and $+2+\delta$ doublets were constrained to 207.4 $\pm$ 0.1 eV

and 205.3 ± 0.1 eV, respectively[1–3] . Furthermore, we constraint the FWHM of the $+4-\delta$ doublet to 1.9 eV. Whereas, the FWHM of the +5 doublet was left as a free parameter and is fitted at 1.7 ± 0.1 eV. The $E_B$ and FWHM of the $+2-\delta$ were also set as free parameters, with $E_B$ fitted at 203.8 ± 0.2 eV and FWHM at 1.5 ± 0.2 eV.[1–3]

## Sc 2p XPS spectra fitting

For Sc 2p XPS spectra, the height ratio between the doublet components (Sc $2p_{1/2}$/Sc $2p_{3/2}$ was constrained to 0.38, with The $E_B$ separation between the components is fixed at 4.47 ± 0.2 eV. Furthermore, the FWHM of the $2p_{1/2}$ component was set to be 1.16 times that of the $2p_{3/2}$ component. These constraints are consistent with the literature.[4]

For all the pre-exposed samples, except for the $Sc_2O_3$, the Sc 2p spectra were fitted with a single doublet. The doublet's $2p_{3/2}$ component is fitted at 402.2 ± 0.2 eV $E_B$, with the FWHM of 1.3 eV. This doublet is ascribed to Sc-atoms in +3 oxidation state, likely in ScOOH and $Sc_2O_3$ chemical states.[4]

For the $Sc_2O_3$ pre-exposed sample, an additional doublet is fitted at 401.0 $E_B$, with FWHM of 1.4 eV. This doublet also corresponds to Sc-atoms in +3 oxidation state in $Sc_2O_3$ chemical state.[4]

Sc 2p spectra of 8 h H*-exposed (for $Sc_2O_3$, 4 h H*-exposed) samples were fitted with a single doublet. The doublet's $2p_{3/2}$ component is fitted at 402.2 − 0.1 eV $E_B$, with the FWHM of 1.8 (+0.2 -0.3) eV. This double corresponds to Sc-atoms in +3 oxidation state[4]

# Comparison of the X-ray Photoelectron Spectroscopy spectra as a function of Θ

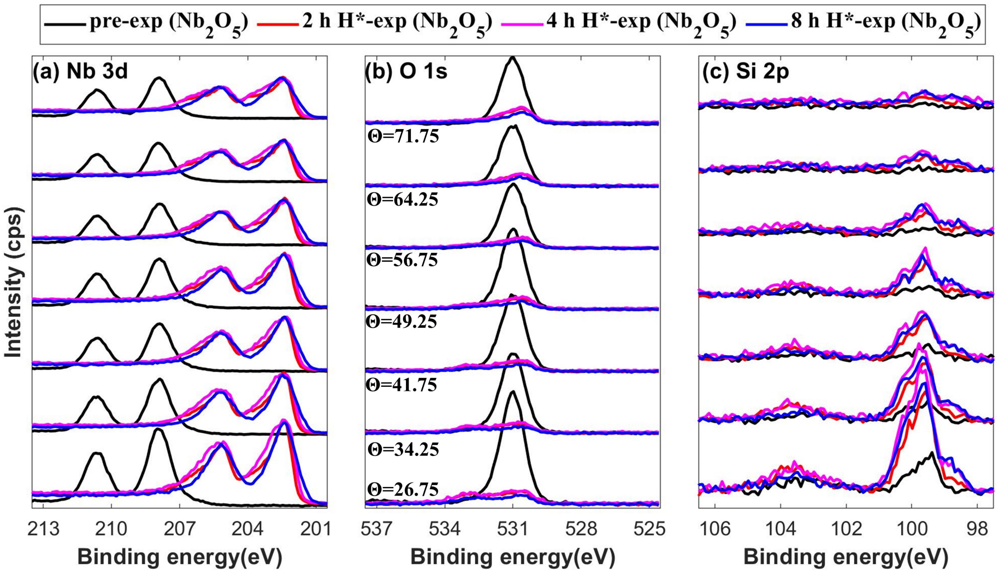


**Figure S9.** XPS spectra of the pre- and post-H*-exposed $Nb_2O_5$ sample; (a) Nb 3d, (b) O 1s, (c) Si 2p. The spectral shapes do not change significantly as a function of Θ. The Si substrate signal is attenuated for more surface sensitive angles.

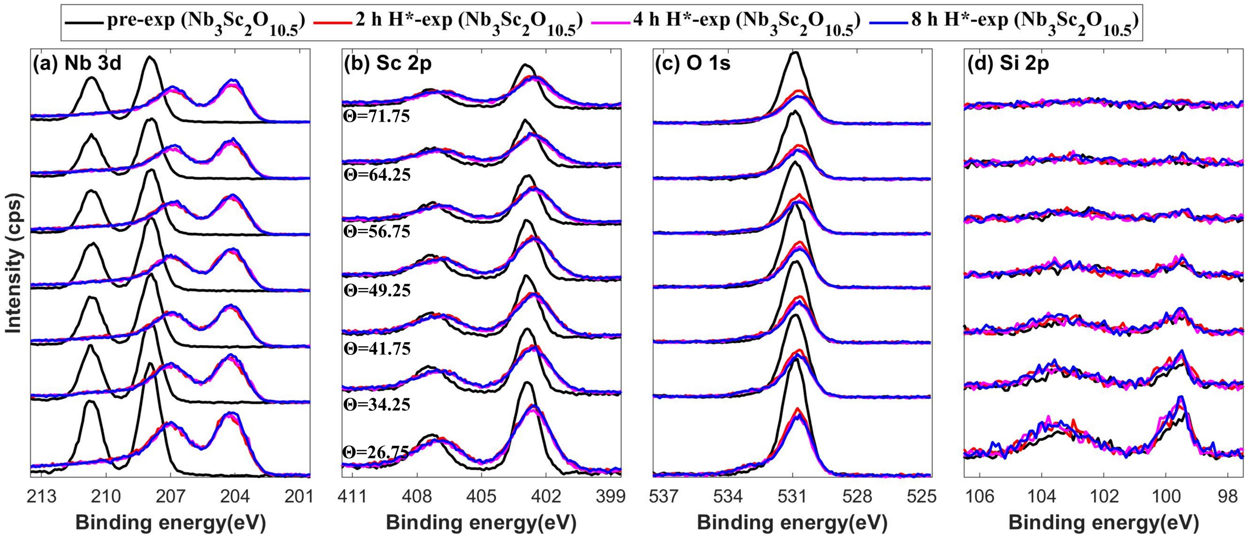


**Figure S10.** XPS spectra of the pre-and post-H*-exposed $Nb_3Sc_2O_{10.5}$ sample; (a) Nb 3d, (b) Sc 2p, (c) O 1s, (d) Si 2p. The spectra show no significant change as a function of Θ.

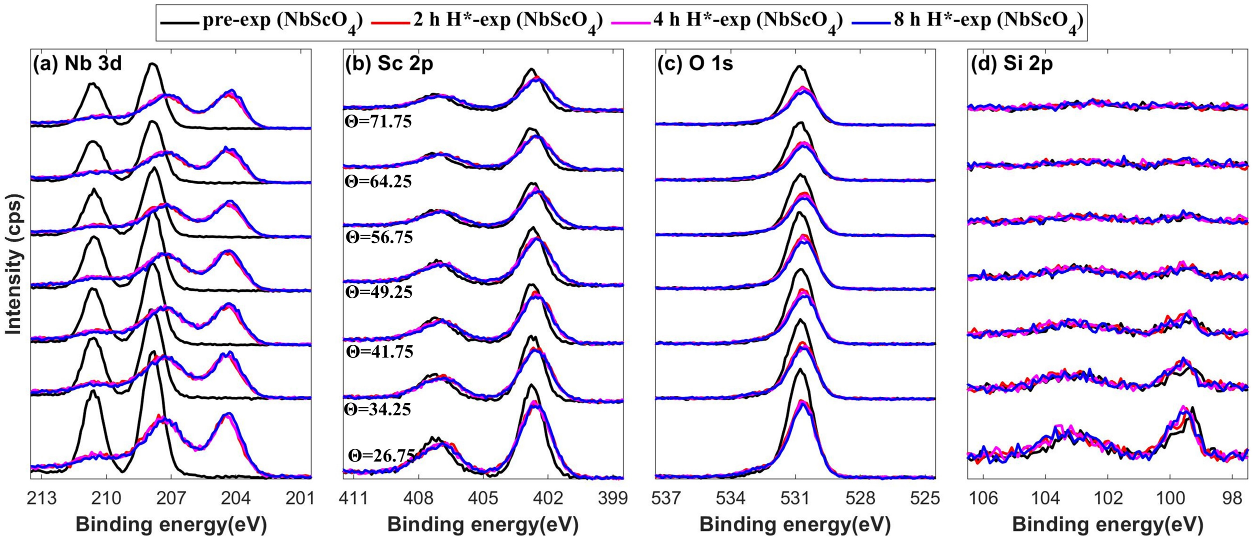


**Figure S11.** XPS spectra of the pre-and post-H*-exposed $NbScO_4$ sample; (a) Nb 3d, (b) Sc 2p, (c) O 1s, (d) Si 2p. Changes in the spectra with Θ are insignificant.

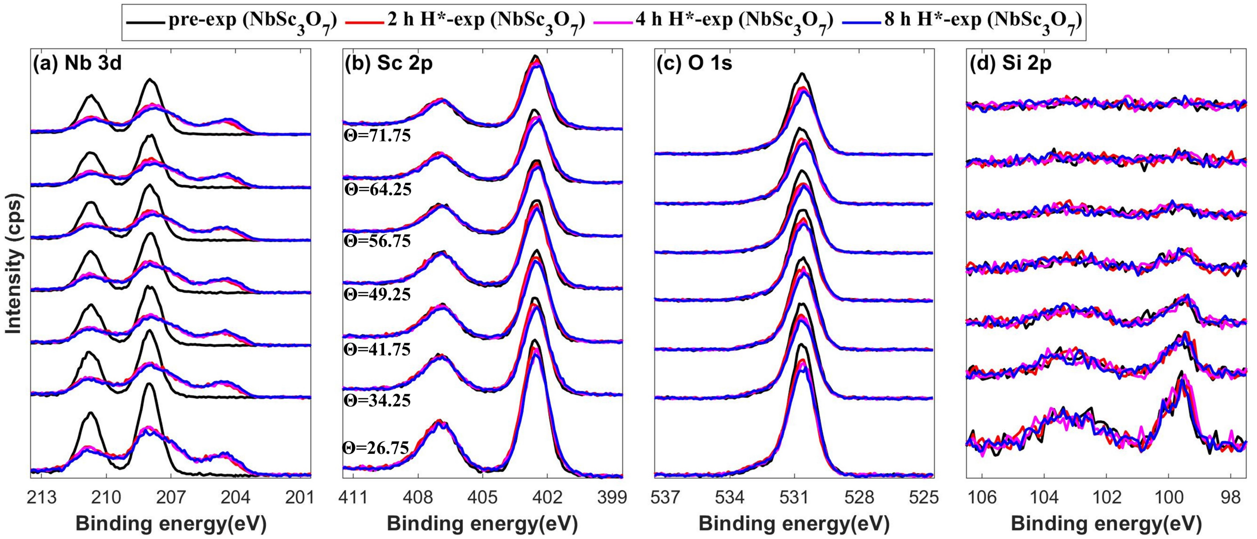


**Figure S12.** XPS spectra of the pre-and post-H*-exposed $NbSc_3O_7$ sample; (a) Nb 3d, (b) Sc 2p, (c) O 1s, (d) Si 2p. The spectral shapes show no significant change with Θ.

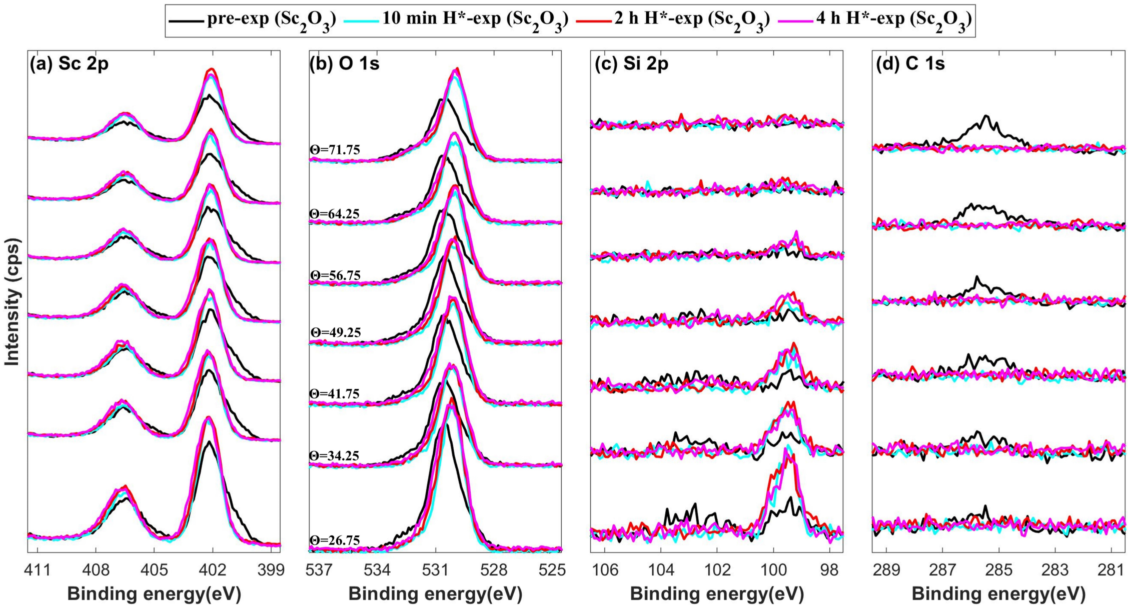


**Figure S13.** XPS spectra of the pre-and post-H*-exposed $Sc_2O_3$ sample; (a) Sc 2p, (b) O 1s, (c) Si 2p, and (d) C 1s. The spectra do not change significantly as a function of $\Theta$.

# Low Kinetic Energy and Valance Band XPS Spectra

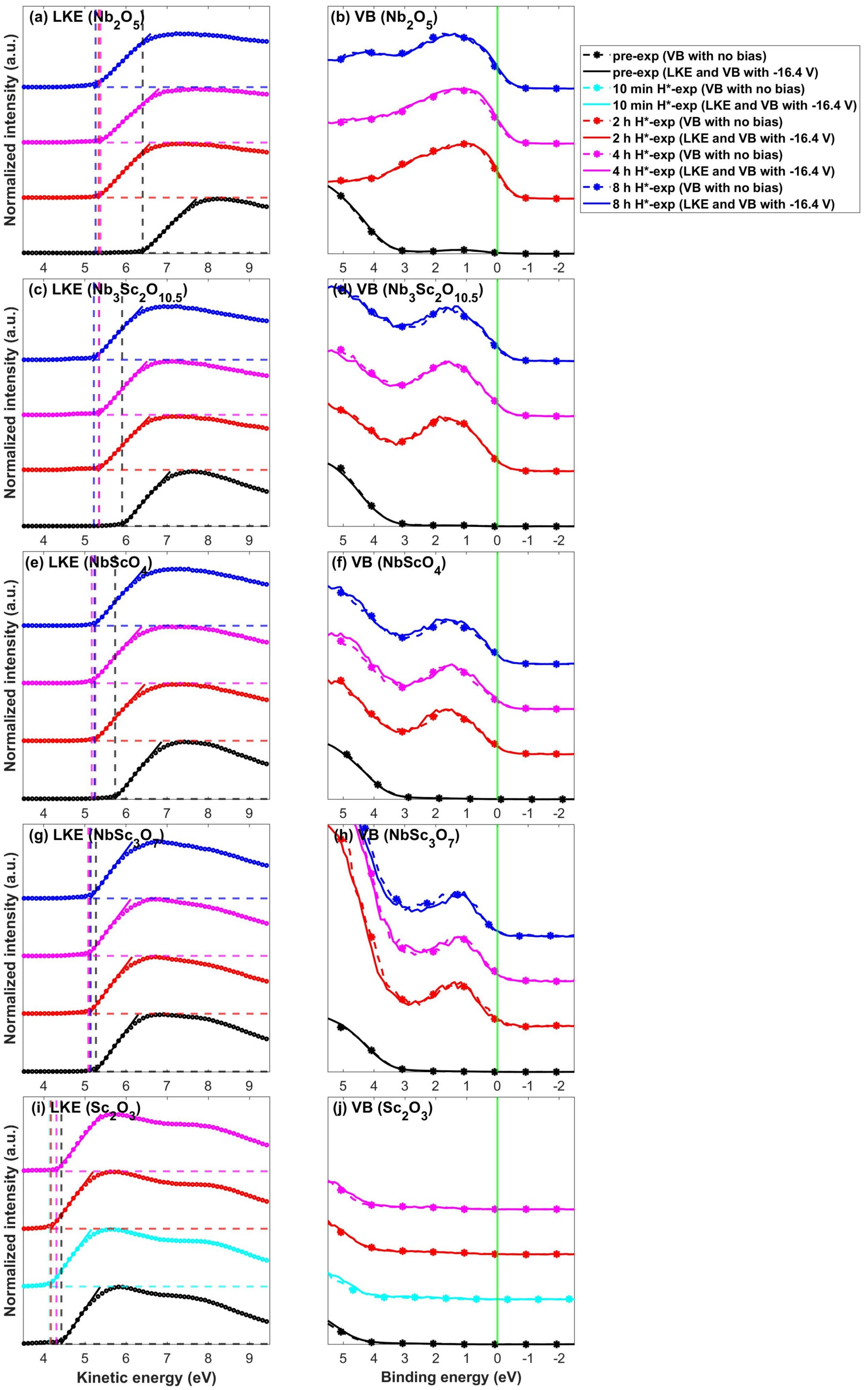


**Figure S14.** LKE and VB spectra of the (a,b) $Nb_2O_5$, (c,d) $Nb_3Sc_2O_{10.5}$, (e,f) $NbScO_4$, (g,h) $NbSc_3O_7$, and (i,j) $Sc_2O_3$ samples. A single secondary electron cut-off suggests that the work function variation across the samples' surfaces is insignificant, consistent with the KPAFM measurements (Figure S16). The VB spectra taken with bias (after shifting the binding energy by the biased voltage) and without bias overlap, suggesting that the samples are sufficiently conductive. The VB spectra of the $Sc_2O_3$ sample collected over a broader range of binding energy are shown in Figure S15.

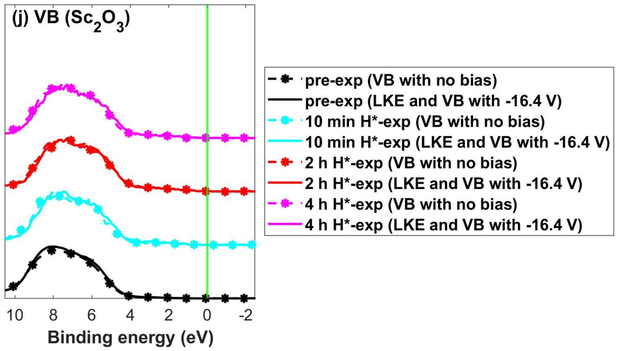


**Figure S15.** VB spectra of the $Sc_2O_3$ sample.

# Work Function measurements via KPAFM

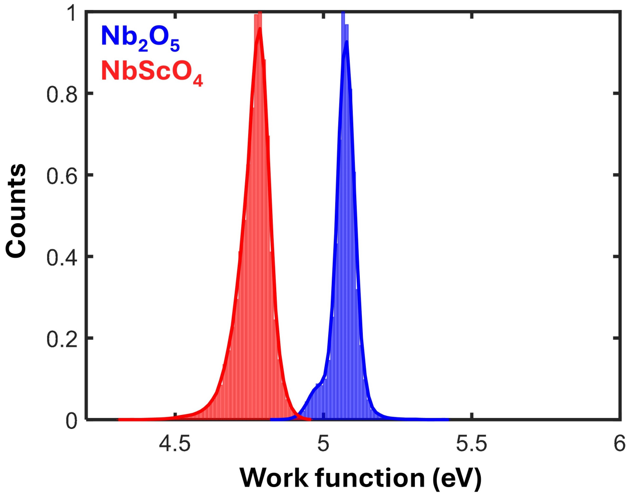


**Figure S16.** The work function of the pre-exposed $Nb_2O_5$ and $NbScO_4$ samples measured via KPAFM. The work function values are in close agreement with those measured via XPS. The Si tip is calibrated against Au pad before and after the work function measurements.

# Deposition rates of the samples

**Table S1. Deposition rates of the samples**

| Sample | Deposition rate (nm/sec) |
|---|---|
| $Nb_2O_5$ | 0.0978 |
| $Nb_3Sc_2O_{10.5}$ | 0.0121 |
| $NbScO_4$ | 0.0097 |
| $NbSc_3O_7$ | 0.0064 |
| $Sc_2O_3$ | 0.0054 |

# Bibliography


(1) Darlinski, A.; Halbritter, J. Angle-resolved XPS studies of oxides at NbN, NbC, and Nb surfaces. *Surface and interface analysis* **1987**, *10*, 223–237.

(2) Weibin, Z.; Weidong, W.; Xueming, W.; Xinlu, C.; Dawei, Y.; Changle, S.; Liping, P.; Yuying, W.; Li, B. The investigation of NbO2 and Nb2O5 electronic structure by XPS, UPS and first principles methods. *Surface and interface analysis* **2013**, *45*, 1206–1210.

(3) Kuznetsov, M.; Razinkin, A.; Shalaeva, E. Photoelectron spectroscopy and diffraction of surface nanoscale NbO/Nb (110) structures. *Journal of Structural Chemistry* **2009**, *50*, 514–521.

(4) Biesinger, M. C.; Lau, L. W.; Gerson, A. R.; Smart, R. S. C. Resolving surface chemical states in XPS analysis of first row transition metals, oxides and hydroxides: Sc, Ti, V, Cu and Zn. *Applied surface science* **2010**, *257*, 887–898.
